# Effect of interferon therapy on quality of life in patients with chronic hepatitis B

**DOI:** 10.1038/s41598-024-51292-4

**Published:** 2024-01-30

**Authors:** Mengdi Zhang, Meijuan Wan, Wen Wang, Shumei Lin, Xi Zhang

**Affiliations:** Department of Infectious Diseases, The First Affiliated Hospital of Xi’an Jiaotong University, Xian, Shaanxi Province China

**Keywords:** Hepatology, Quality of life

## Abstract

Interferon therapy is the most effective treatment for achieving clinical cure in chronic hepatitis B (CHB) patients. However, the treatment outcomes of interferon therapy are uncertain, multiple side effects can occur during treatment, and the treatment is expensive. Although these characteristics may affect patients’ quality of life, research examining this topic is limited. We used a cross-sectional design to examine 100 CHB patients receiving interferon, 100 receiving nucleoside/nucleotide analogues, and 87 receiving non-antiviral treatment. Characteristic information, the Hepatitis B Quality of Life Instrument, Connor Davidson Resilience Scale, and Work Productivity and Activity Impairment Questionnaire were used to collect information. We found that quality of life in the interferon treatment group was higher than that in the non-antiviral treatment and nucleoside/nucleotide analogue treatment groups (*p *< 0.05). The factors influencing quality of life were resilience, presenteeism, hair loss, and antiviral treatment (*p *< 0.05). Although interferon therapy has some potential side effects, the results suggested that it did not negatively affect quality of life. Overall, interferon therapy did not have a major impact on CHB patients’ daily lives and work.

## Introduction

Chronic hepatitis B (CHB) is a systemic infectious disease caused by the hepatitis B virus (HBV) with liver damage as the primary cause^1^. According to World Health Organization data released in 2019, the global prevalence of CHB infection was approximately 3.5%, with 820,000 people dying from liver failure, cirrhosis, and hepatocellular carcinoma caused by HBV infection^2^. Clinical cure is the ideal treatment target recommended in the latest guidelines for the prevention and treatment of CHB^1,2^. A previous study reported that patients who are clinically cured can safely discontinue drug treatment for an extended period of time, with a reported incidence of hepatocellular carcinoma of 0%–1% within 5 years^3^. Immunomodulators such as pegylated interferon alpha (PEG-IFNα), are currently considered to be the most effective antiviral therapy for clinical cure^1^. However, the uncertainty of the outcomes of interferon therapy, coupled with a substantial incidence of adverse reactions during treatment, the potential for relapse even after achieving clinical cure, the long treatment period, and the high cost, may all have an impact on patients’ physiological, psychological, and social functioning^4–6^. Previous studies of interferon therapy for CHB patients mainly focused on therapeutic efficacy, prognosis, and predictive indicators^7^, with little attention paid to psychosocial aspects.

Over many years, the traditional medical model has changed to the bio-psycho-social medical model, and medical research has gradually begun to pay attention to the relationships between human mental health and the development and outcomes of diseases. Therefore, the psychosocial aspects of patients undergoing interferon therapy for CHB needs attention. Quality of life is a concept that integrates biomedical, sociological, and psychological aspects to provide a more comprehensive picture of health, and to assess the effectiveness of treatments^8,9^. Previous studies have shown that quality of life decreases in patients with chronic hepatitis C during interferon therapy^10,11^. However, it is not clear whether interferon therapy affects quality of life in patients with CHB during interferon therapy. This current study aimed to explore the relationship between interferon therapy and quality of life in patients with CHB.

## Methods

### Study design and sample

From April 2022 to May 2023, a convenience sampling method was used to recruit 100 CHB patients who received interferon therapy, and 187 non-interferon treated CHB patients in the outpatient clinic of the Infection Department of a Grade III Level A hospital which annually receives nearly 70,000 outpatients with CHB. The sample size was calculated using PASS15.0. In previous study^12^ assessing the quality of life in patients with CHB and pre-investigation of quality of life in patients with CHB treated with interferon, the quality of life scores of CHB patients were (1952.97 ± 742.19) points and (2274.59 ± 677.26) points. We selected “Two-Sample T-Tests” as the calculation method. When α = 0.05 (bilateral), power = 0.80, and the expected effect size (Cohen’s d) = 0.5, we calculated that 78 cases were needed in each group. The sample size was expanded by 10% to 87 cases in each group.

With the consent of the hospital administration, the researchers conducted an investigation in the outpatient infection department. The purpose of the study was explained to participants before the investigation. After obtaining participants’ consent, data were collected using an online method powered by www.wjx.cn. To control the quality of questionnaire responses, all patients filled out the questionnaire under standardised guidance. If the time taken to answer the questionnaire was less than 6 min, the questionnaire was considered to be of low quality.

Inclusion criteria: (1) patients met the relevant diagnostic criteria of the Chronic Hepatitis B Prevention and Treatment Guidelines^1^; (2) patients in the interferon group received PEG-IFNα treatment for ≥ 1 month; (3) patients volunteered to participate in this study and provided written informed consent. Exclusion criteria: (1) patients had other types of hepatitis virus infectious; (2) patients had cirrhosis, hepatocellular carcinoma, major diseases of the heart, brain, lungs, or other organs; (3) the quality of patients’ questionnaire responses was poor, or they experienced difficulties in communication, reading, writing, or comprehension.

### Measurements

*Socio-demographic and health status.* A self-designed questionnaire was used, including gender, age, education level, occupation, marital status, type of medical insurance, hepatitis B history, family history of hepatitis B, side effects, hepatitis B surface antigen (HBsAg), hepatitis B e antigen (HBeAg) and hepatitis B virus DNA (HBV DNA).

*The Hepatitis B Quality of Life Instrument (HBQOL)*. The Hepatitis B Quality of Life Instrument (version 1.0, HBQOL V.1.0) was produced by Spiegel in 2007^13,14^. The HBQOL has a total of 31 items, and seven dimensions: psychological well-being, anticipation, vitality, stigma, vulnerability, transmission, viral response, each item is scored on a 5-point scale, and the conversion formula of the score is: (5-selected option) multiplied by 25. The higher the score, the higher the quality of life of the CHB patients. Cronbach’s α coefficient was 0.960.

*Connor Davidson Resilience Scale (CD-RISC)*. The Connor Davidson Resilience Scale (CD-RISC) was produced by Connor and Davidson in 2003^15^. In this study, the Chinese version of the CD-RISC was used, which was translated and adapted by Yu and Zhang in 2007^16^, which included 25 items in three dimensions: tenacity, strength, and optimism. A 5-point Likert scale method was adopted, and Cronbach’s α coefficient was 0.937.

*Work Productivity and Activity Impairment Questionnaire Specific Health Problem* (WPAI-SHP). The WAPI-SHP was compiled by Reilly and colleages^17^ in 1993. This questionnaire consists of six questions and is used to determine employment status, time of absence caused by illness, time of absence for other reasons, actual working time, degree of impact of illness on work productivity at work, and degree of impact of illness on activities outside work. The WAPI-SHP has four components, providing scores for absenteeism, presenteeism, activity impairment, and overall work impairment. Higher scores indicate greater negative impact.

### Statistical analysis

Continuous and categorical variables were reported as mean ± standard deviation (SD) or frequencies (percentages). Demographic data and health data were analyzed using descriptive statistics. The quality of life, general information, mental resilience, and working ability of the interferon treatment group, non-antiviral treatment group, and nucleoside/nucleotide analogues (NAs) treatment group were compared using *χ*^*2*^ tests, analysis of variance, and the Kruskal–Wallis H test. Multiple linear regression analysis was performed to examine the influencing factors of quality of life in CHB patients. All analyses were performed using SPSS 22.0 Statistics (IBM Corporation, Armonk New York, USA). The level of statistical significance was set at *p *< 0.05.

### Ethical approval and consent to participate

The study protocol was approved by the Biomedical Ethics Committee of Xi’an Jiaotong University Health Science Center (Approval No. 2022–1430). Participation was voluntary, and informed consent was given at the top of the questionnaire. Participants anonymously answered questions that were consistent with their agreement to participate and agreed to the publication of the results. The investigation conformed with the principles outlined in the Declaration of Helsinki (Br Med J 1964;ii:177).

## Results

A total of 300 questionnaires were collected. 13 participants were excluded from the questionnaire because of low quality responses, and the effective recovery rate was 95.7%.

### Demographic analysis of CHB patients

Demographic analysis of CHB patients receiving interferon treatment, NAs treatment, and non-antiviral therapy revealed no significant differences in gender, age, education, occupation, marital status, medical insurance, family history, or hepatitis B history (*p *> 0.05), as shown in Table 1.

**Table 1:** Comparison of Demographic of CHB patients in the interferon treated group, non-antiviral treatment group and the NAs treated group.

	Interferon treatment Mean ± SD or n (%) (N = 100)	Non-antiviral treatment Mean ± SD or n (%) (N = 100)	NAs treatment Mean ± SD or n (%) (N = 87)	*χ*^2^(*Z*)	*P*
Age (years)	37.90 ± 7.17	37.04 ± 8.26	36.92 ± 7.28	0.48^a^	0.620
Gender				1.95	0.377
Man	66 (66%)	58 (58%)	58 (66.7%)		
Male	34 (34%)	42 (42%)	29 (33.3%)		
Educational level				7.97	0.093
High school and below	25 (25%)	33 (33%)	29 (33.3%)		
Technical secondary and junior college	24 (24%)	17 (17%)	8 (9.2%)		
Bachelor degree or above	51 (51%)	50 (50%)	50 (57.5%)		
Profession				1.16	0.979
Peasant	14 (14%)	16 (16%)	13 (14.9%)		
Worker	11 (11%)	10 (10%)	11 (12.6%)		
Company employee	40 (40%)	36 (36%)	35 (40.2%)		
Others	35 (35%)	38 (38%)	28 (32.2%)		
Marital status				0.76	0.683
Married	84 (84%)	83 (83%)	69 (79.3%)		
Spinsterhood	16 (16%)	17 (17%)	18 (20.7%)		
Health insurance				0.49	0.782
Have	83 (83%)	86 (86%)	75 (86.2%)		
Not	17 (17%)	14 (14%)	12 (13.8%)		
Family history of hepatitis B				1.11	0.574
Have	67 (67%)	65 (65%)	35 (40.2%)		
Not	33 (33%)	35 (35%)	52 (59.8%)		
History of hepatitis B (years)	17.65 ± 9.57	14.68 ± 9.48	16.4 ± 10.07	4.03^a^	0.133

^a^Indicates the* Z* value.

### Factors influencing quality of life in CHB patients

Univariate analysis showed that the factors affecting the quality of life of CHB patients were as follows: level of education, profession, health insurance, hair loss, antiviral treatment, HBsAg, absenteeism, presenteeism, overall work impairment, activity impairment, resilience, tenacity, strength, and optimism (*p *< 0.05), as shown in Tables 2 and 3. Continuous variables, such as quality of life, resilience, presenteeism, and HBsAg were assigned as measured values. Binary variables were assigned, with a value of 1 for hair loss and 0 for no hair loss. Dummy variables were introduced to describe the multi-categorical variable “antiviral treatment,” with a value of “0,0” for interferon treatment, “1,0” for NAs treatment, and “0,1” for non-antiviral treatment. The quality-of-life score of CHB patients was used as the dependent variable, and level of education, profession, and health insurance were used as independent variables. The regression model had statistical significance *F* = 21.516, (*p *< 0.001), and adjusted *R*^*2*^ = 0.315. The results indicated that the factors affecting the quality of life of CHB patients were resilience, presenteeism, hair loss, and antiviral treatment (*p *< 0.05), as shown in Table 4.

**Table 2:** Univariate analysis of factors affecting quality of life.

	Quality of life Mean ± SD (N = 287)	*t/Z*	*P*
Gender		− 0.899	0.369
Man	2128.02 ± 733.67		
Male	2047.86 ± 716.71		
Educational level		6.935^a^	0.001
High school and below	1870.11 ± 809.77		
Technical secondary and junior college	2287.76 ± 703.25		
Bachelor degree or above	2169.04 ± 653.72		
Profession		9.601^a^	< 0.001
Peasant	1584.88 ± 687.74		
Worker	2170.31 ± 743.37		
Company employee	2140.99 ± 624.01		
Others	2248.27 ± 758.12		
Marital status		− 0.438	0.662
Married	2089.94 ± 730.18		
Spinsterhood	2139.22 ± 719.45		
Health insurance		2.670	0.008
Have	2146.31 ± 708.09		
Not	1828.49 ± 782.96		
Family history of hepatitis B		0.933	0.352
Have	2045.15 ± 756.06		
Not	2128.67 ± 711.00		
HBVDNA (IU/ml)		1.755^a^	0.175
< 100 or < 10	2150.90 ± 704.14		
10 ~ 10^4^	2034.74 ± 769.36		
> 10^4^	1884.26 ± 773.86		
HBeAg		− 1.285	0.200
(+)	1961.36 ± 837.57		
(−)	2119.72 ± 714.00		
Fever		− 0.353	0.725
Yes	2022.73 ± 753.70		
No	2101.72 ± 727.46		
Weakness		− 1.408	0.160
Yes	1999.03 ± 688.91		
No	2135.24 ± 739.08		
Joint dnd muscle pain		− 0.394	0.694
Yes	2050.00 ± 602.36		
No	2104.60 ± 741.78		
Dizziness		− 1.461	0.145
Yes	1896.00 ± 590.85		
No	2118.03 ± 737.02		
Nausea and anorexia		− 0.301	0.763
Yes	2066.03 ± 675.35		
No	2103.84 ± 736.27		
Hair loss		− 2.3556	0.019
Yes	1890.28 ± 546.61		
No	2146.99 ± 755.88		
Gingiva bleeding		− 0.929	0.354
Yes	1998.08 ± 689.02		
No	2114.52 ± 733.19		
Skin rash		0.577	0.565
Yes	2186.90 ± 619.20		
No	2091.73 ± 735.70		
Antiviral treatment		10.014^a^	< 0.001
Interferon	2352.25 ± 564.35		
NAs	1987.64 ± 714.27		
Non-antiviral	1941.75 ± 815.92		

^a^Represents *Z* value.

**Table 3:** Univariate analysis of factors affecting quality of life.

	Quality of life	
	*r*	*P*
Age (years)	0.002	0.979
History of hepatitis B (years)	− 0.089	0.134
HBsAg (IU/ml)	− 0.136	0.036
Absenteeism	0.024	0.739
Presenteeism	− 0.270	< 0.001
Overall work impairment	− 0.231	0.001
Activity impairment	− 0.257	< 0.001
Resilience	0.412	< 0.001
Tenacity	0.393	< 0.001
Strength	0.411	< 0.001
Optimism	0.312	< 0.001

**Table 4:** Multivariate linear regression analysis of factors affecting quality of life.

Predication variable	*B*	95% CI	*SE*	*β*	*t*	*P*
Constant	2140.794	(1734.203,2547.385)	206.199	−	10.382	< 0.001
Resilience	10.702	(5.870,15.533)	2.137	0.268	4.367	< 0.001
Presenteeism	− 10.821	(− 15.034, − 6.607)	2.137	− 0.391	− 5.064	< 0.001
Hair loss	− 272.766	(− 475.619, − 69.914)	102.875	− 0.157	− 2.651	0.009
NAs treated	− 731.786	(− 966.565, − 497.007)	119.066	− 0.483	− 6.146	< 0.001
Non-antiviral treatment	− 431.567	(− 619.764, − 243.369)	95.443	− 0.288	− 4.522	< 0.001

### Effect of different antiviral therapy on quality of life in patients with CHB

**Figure 1:**
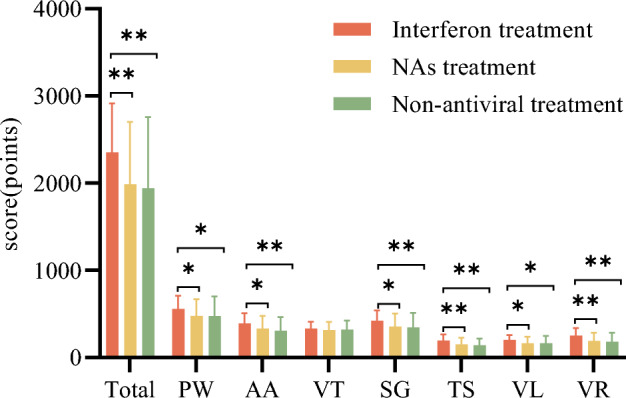
Comparison of quality of life of CHB patients in interferon treated group, untreated group and NAs treated group. The total score for quality of life (Total) Psychological Well-Being (PW), Anticipation (AA), Vitality (Vitality), Stigma (SG), Vulnerability (VL), Transmission (TS), Viral Response (VR). *P < 0.05, **P < 0.001.

CHB patients treated with interferon scored higher for quality of life, psychological well-being, anticipation, stigma, vulnerability, transmission, and viral response compared with non-antiviral and NAs treatment patients (*p *< 0.05), as shown in Table 5 and Fig. 1.

**Table 5:** Comparison of quality of life of CHB patients in interferon treated group, untreated group and NAs treated group.

	Interferon treatment Mean ± SD (N = 100)	Non-antiviral treatment Mean ± SD (N = 100)	NAs treatment Mean ± SD (N = 87)	*F*	*P*
Quality of life	2325.25 ± 564.35	1941.75 ± 815.92^a^	1987.64 ± 714.27^b^	10.014	< 0.001
Psychological well-being	556.75 ± 151.58	476.50 ± 225.26^a^	478.74 ± 191.14^b^	5.563	0.004
Anticipation	392.00 ± 115.74	308.25 ± 155.54^a^	330.17 ± 146.77^b^	9.549	< 0.001
Vitality	332.00 ± 78.66	322.50 ± 101.34	316.09 ± 93.46	0.719	0.488
Stigma	421.25 ± 121.15	345.00 ± 166.14^a^	354.89 ± 151.20^b^	7.827	< 0.001
Transmission	196.50 ± 69.81	140.75 ± 78.55^a^	151.44 ± 78.07^b^	8.019	< 0.001
Vulnerability	201.00 ± 56.29	165.75 ± 83.91^a^	164.65 ± 73.59^b^	15.932	< 0.001
Viral response	252.75 ± 84.68	183.00 ± 103.41^a^	191.67 ± 94.67^b^	10.014	< 0.001

^a^Indicates that *P *< 0.05 was compared between Interferon treated group and Non-antiviral treatment.

^b^Indicates that *P *< 0.05 was compared between Interferon treated group and NAs treated group.

## Discussion

Our study found that CHB patients treated with interferon had higher quality of life compared with those treated with antiviral therapy and NAs. Interferon therapy may be associated with an improvement in quality of life for CHB patients.

There are several possible explanations for the higher quality of life of CHB patients treated with interferon. First, interferon therapy has been reported to induce negative HBV DNA conversion, HBeAg serological conversion, and negative HBsAg conversion to achieve clinical cure; CHB patients who achieve clinical cure are reported to have a lower incidence of liver cirrhosis and hepatocellular carcinoma^18^, which is a key factor affecting the psychosocial health of CHB patients^19^. A previous study reported that CHB patients who exhibited better responses during antiviral therapy had a higher quality of life^20^. Second, decreased quality of life caused by HBV infection is reported to be primarily related to impacts on mental health and social health, with little impact on physical health^21^. Although people with CHB have fewer physical symptoms and are more stable, the risk of disease progression and severe psychosocial harms^19,22,23^ (e.g., fear of transmitting to others, disclosure of HBV infection, discrimination, and anxiety, depression, etc.) can compromise their quality of life. A multicenter study conducted in Turkey^24^ reported that CHB patients receiving antiviral therapy exhibited more severe disease symptoms but had better mental health and social functioning and less anxiety compared with CHB patients who did not receive antiviral therapy. In the current study, although interferon therapy caused some adverse reactions, quality of life was still higher for CHB patients receiving interferon therapy compared with those that received non-interferon treatment, indicating that interferon treatment had a beneficial effect on psychosocial health in CHB patients.

In the current study, the factors affecting the quality of life of CHB patients were resilience, presenteeism, hair loss, and antiviral treatment. The resilience score of CHB patients treated with interferon in the current study was 64.6 ± 16.26 points, which was slightly higher than that that reported in a previous study (61.64 ± 15.36)^25^. Previous studies have reported that patients with a high level of resilience tend to have a positive attitude and exhibit coping behaviors, and can manage the stress and impact of their disease in a positive way, leading to improved quality of life in a wide range of chronic diseases^16,26–28^.

As an important aspect of people’s social lives, work is a major factor in social health, which is one of the components of quality of life^29,30^. Some of the items in the quality- of-life scale used in the current study were directly or indirectly related to the impact of current health conditions on work, such as “fear that having hepatitis B will be discovered by someone, such as a supervisor.” Decreased work productivity may reflect a decrease in social health, which can affect mental health, thus further affecting the quality of life.

Loss of hair occurring in any disease can negatively impact quality of life, and is often associated with loss of self-esteem and psychosocial problems^31^. Hair loss is one of the common side effects of interferon therapy^32,33^, and the incidence of hair loss in the interferon treatment group in our study was 27%. The impact on life and work, and potential side effects, are significant reasons for CHB patients’ dissatisfaction with interferon therapy^34^. However, the side effects of interferon treatment, including interferon-induced hair loss, are temporary, and most people regain growth within 3 to 6 months after stopping interferon. Therefore, the impact of potential hair loss on daily life should not be a major concern for CHB patients.

The results of the current study indicated a positive association between interferon therapy and quality of life in CHB patients. Improvement in quality of life was found to be influenced by resilience, presenteeism, and hair loss. Despite potential temporary side effects, the results indicated that interferon therapy alleviated psychological and social distress, contributing to enhanced quality of life. Thus, the impact of interferon therapy on the daily lives and work of CHB patients does not appear to be a major cause for concern.

## Limitations

Because of time constraints, the present study was a cross-sectional investigation of CHB patients treated with interferon. In future, longitudinal studies should be carried out to further determine the impact of interferon treatment on CHB patients’ quality of life by examining changes in quality of life over time during treatment.

## Data Availability

All data generated or analysed during this study are included in this published article. The datasets generated and/or analysed during the current study are available from the corresponding author on reasonable request.
